# Characterizing the impact of MnO_2_ addition on the efficiency of Fe^0^/H_2_O systems

**DOI:** 10.1038/s41598-021-89318-w

**Published:** 2021-05-07

**Authors:** Viet Cao, Ghinwa Alyoussef, Nadège Gatcha-Bandjun, Willis Gwenzi, Chicgoua Noubactep

**Affiliations:** 1 Faculty of Natural Sciences, Hung Vuong University, Nguyen Tat Thanh Street, Viet Tri, Phu Tho 35120 Vietnam; 2 Angewandte Geologie, Universität Göttingen, Goldschmidtstraße 3, 37077 Göttingen, Germany; 3 Faculty of Science, Department of Chemistry, University of Maroua, BP 46, Maroua, Cameroon; 4 Biosystems and Environmental Engineering Research Group, Department of Agricultural and Biosystems Engineering, University of Zimbabwe, P.O. Box MP167, Mt. Pleasant, Harare Zimbabwe; 5 Centre for Modern Indian Studies (CeMIS), Universität Göttingen, Waldweg 26, 37073 Göttingen, Germany; 6 Department of Water and Environmental Science and Engineering, Nelson Mandela African Institution of Science and Technology, P.O. Box 447, Arusha, Tanzania

**Keywords:** Environmental chemistry, Environmental social sciences, Materials science

## Abstract

The role of manganese dioxide (MnO_2_) in the process of water treatment using metallic iron (Fe^0^/H_2_O) was investigated in quiescent batch experiments for t ≤ 60 d. MnO_2_ was used as an agent to control the availability of solid iron corrosion products (FeCPs) while methylene blue (MB) was an indicator of reactivity. The investigated systems were: (1) Fe^0^, (2) MnO_2_, (3) sand, (4) Fe^0^/sand, (5) Fe^0^/MnO_2_, and (6) Fe^0^/sand/MnO_2_. The experiments were performed in test tubes each containing 22.0 mL of MB (10 mg L^−1^) and the solid aggregates. The initial pH value was 8.2. Each system was characterized for the final concentration of H^+^, Fe, and MB. Results show no detectable level of dissolved iron after 47 days. Final pH values varied from 7.4 to 9.8. The MB discoloration efficiency varies from 40 to 80% as the MnO_2_ loading increases from 2.3 to 45 g L^−1^. MB discoloration is only quantitative when the operational fixation capacity of MnO_2_ for Fe^2+^ was exhausted. This corresponds to the event where adsorption and co-precipitation with FeCPs is intensive. Adsorption and co-precipitation are thus the fundamental mechanisms of decontamination in Fe^0^/H_2_O systems. Hybrid Fe^0^/MnO_2_ systems are potential candidates for the design of more sustainable Fe^0^ filters.

## Introduction

Water pollution has a significant negative impacts on ecological and human health. These impacts are expected to substantially increase in the coming decades due to: (1) increased urbanization, (2) increased industrialization, and (3) climate change^1–3^. The low-income communities who are spatially scattered, and economically disadvantaged are most impacted by water pollution or lack of safe drinking water^2–4^. Thus, low-income communities need affordable, efficient and applicable technologies for their drinking water supply. Water filtration using sand filters amended with metallic iron (Fe^0^ filters) has been demonstrated to be such an affordable and applicable technology^5–11^.

The use of Fe^0^ for environmental remediation and water treatment has boomed over the past 30 years^12–21^. Fe^0^ is mainly regarded as an environmentally friendly reducing agent (E^0^ =  − 0.44 V)^12,20,21^. It has been successfully applied for the removal of a myriad of environmental pollutants from groundwater and wastewater^12,15,20^. Fe^0^ was initially used as micro-sized granular material^12^. Efforts to alleviate material passivation has prompted to develop granular bimetallics^6–8^, granular composites^13,15^, granular material mixtures (e.g. Fe^0^/Fe_3_O_4_, Fe^0^/FeS_2_, Fe^0^/MnO_2_)^20,21^, and their nano-sized counterparts^16–19^. There is increasing interest on using granular Fe^0^ for the design of decentralized drinking water supply units^6,10^. In particular, the ternary mixture Fe^0^/MnO_2_/sand seems very promising^21^.

Fe^0^ filters are a special case of "metal corrosion in porous media"^22^. This process has two key characteristics^23,24^: (1) the time-dependent decrease of the reaction kinetics of iron corrosion (“reactivity loss”), and (2) the progressive decrease of the hydraulic conductivity (permeability loss) due to the initial porosity being filled by in-situ generated iron oxides and hydroxides. In other words, the design of sustainable Fe^0^ filters should account for the long-term corrosion rate of the used Fe^0^ specimens. A mathematical modelling has enabled a spatial solution of the issue of permeability loss^24,25^. It is established that only hybrid Fe^0^ filters containing Fe^0^ and other aggregates (e.g. Fe^0^/Fe_3_O_4_, Fe^0^/gravel, Fe^0^/MnO_2_, Fe^0^/sand) are sustainable because non-expansive aggregates are not contributing to porosity/permeability loss^24^. Yet how fast the porosity decreases as a function of time is an open issue and has received very little attention^26–28^. However, it is known from the broad corrosion literature that the corrosion kinetics of metals, including Fe^0^ under environmental conditions, is neither constant nor linear^22,29^. Therefore, it is impossible to predict the service life of a Fe^0^ filter without more accurate data on the corrosion rate, which is material-dependent^29,30^. It is thus not surprising that all models presented for the prediction of the operation of Fe^0^-based permeable reactive barriers were not successful^26,27,31^. The present work is devoted at qualitatively characterizing the efficiency of Fe^0^/H_2_O systems for water treatment as influenced by the presence of MnO_2_. The methylene blue discoloration (MB method) developed in earlier studies is used^21,31–35^. In particular, MnO_2_ is used to control the availability of 'free' iron corrosion products (FeCPs).

Since the seminal work of Bischof^5^, Fe^0^, gravel/sand and MnO_2_ have been used in Fe^0^ filters. Fe^0^ is electrochemically oxidized by water to generate Fe^2+^ and H_2_ (Eq. 1). The original device of Bischof contained a layer of pyrulosite (a MnO_2_ mineral) after the Fe^0^/gravel layer^5^. Pyrulosite acts as a Fe^2+^ scavenger (Eq. 2) to lower the iron concentration in filtered water. Equation (2) represents the reductive dissolution of MnO_2_ (or MnO_x_) which can be regarded as a stand alone branch of environmental geochemistry^36–38^.

1 $$ {\text{Fe}}^{0} + 2{\text{H}}^{ + } \Rightarrow {\text{Fe}}^{2 + } {\text{ + H}}_{2} $$

2 $$ 2{\text{Fe}}^{2 + } {\text{ + MnO}}_{2} + 4{\text{H}}^{ + } \Rightarrow {\text{Mn}}^{2 + } + 2{\text{Fe}}^{3 + } + 2{\text{H}}_{2} {\text{O}} $$

In other words, Fe^2+^ is oxidized at the surface of MnO_2_ and is ideally not transported out of the filter. It is obvious that the Fe^2+^ scavenging efficiency of the pyrulosite layer depends on the amount used and its intrinsic reactivity.

During the past two decades, MnO_2_ and Fe^0^ have been mixed to enhance the efficiency of Fe^0^/H_2_O systems for the removal of various contaminants, including uranium and radon^39^, diclofenac^40,41^, heavy metals^42^, methylene blue^21,32–35,43^, chromium^44,45^, arsenic^46^, and tetracycline^47^. In these efforts, Fe^0^/MnO_2_ composites were also used^6,46,48,49^ and enhanced contaminant removal explained by electrochemical cells between Fe^0^ and MnO_2_ like in some Mn-rich Fe^0^ specimens^21,50,51^. On the other hand, Dong et al.^47^ tested the sequence MnO_2_–Fe^0^ (MnO_2_ layer before Fe^0^) and also reported on enhanced tetracycline removal compared to the single-aggregate-systems (e.g., Fe^0^ and MnO_2_). Clearly, information rationalizing the positive impact of MnO_2_ on the efficiency of Fe^0^/H_2_O systems is confusing. The methylene blue method^32,33,52^ can help in elucidating the mechanisms of contaminant removal in Fe^0^/H_2_O systems.

Methylene blue (MB, a cationic dye) has been demonstrated to be an indicator of the reactivity of the Fe^0^/H_2_O system^21,32–35,52^. Its suitability is based on its cationic nature and its differential affinity to positively charged iron oxide surfaces and negatively charged surface of sand^21,53^. Using MB as a reactivity indicator has improved our knowledge on the Fe^0^/H_2_O system during the past decade^35,43,52,54–58^. In particular, Miyajima and Noubactep^35^ utilized the systems used in this study and reported on lowered MB discoloration in MnO_2_ amended Fe^0^/H_2_O systems for 14 d.

The objective of this study is to investigate the impact of various amounts of three different MnO_2_ on the efficiency of Fe^0^/H_2_O systems for MB discoloration. The specific objective is to confirm the suitability of ‘MB discoloration’ as powerful tool for the characterization of processes in Fe^0^/H_2_O systems while using MnO_2_ and sand to control the availability of ‘free’ FeCPs. The extent of MB discoloration is characterized using five different systems: (1) Fe^0^ alone, (2) sand alone, (3) Fe^0^/sand, (4) Fe^0^/MnO_2_, and (5) Fe^0^/MnO_2_/sand for up to 60 days. A comparison of the results from the five systems will provide critical information on the contaminant removal mechanisms and the role of MnO_2_.

## Materials and methods

### Solutions

The used methylene blue (MB – Basic Blue 9 from Merck) was of analytical grade. The working solution was 10.0 mg L^−1^ prepared by diluting a 1000 mg L^−1^ stock solution. The stock solution was prepared by dissolving accurately weighted MB in tap water. The use of tap water rather than deionised water was motivated by the fact that tap water is closer to natural water in its chemical composition. The MB molecular formula is C_16_H_18_N_3_SCl corresponding to a molecular weight of 319.85 g. MB was chosen in this study because of its well-known strong adsorption onto solids^32,53^.

### Solid materials

#### Metallic iron (Fe^0^)

The used Fe^0^ material was purchased from iPutech (Rheinfelden, Germany). The material is available as filings with a particle size between 0.3 and 2.0 mm. Its elemental composition as specified by the supplier was: C: 3.52%; Si: 2.12%; Mn: 0.93%; Cr: 0.66% (balanced by Fe). The material was used without any further pre-treatment. Fe^0^ was proven as a powerful discoloration agent for MB given that discoloration agents in the form of FeCPs are progressively generated in-situ^54,55^.

#### Manganese dioxide (MnO_2_)

Three natural MnO_2_-bearing minerals were tested: (1) Manganit (Ilfeld/Harz; Thüringen/Germany), (2) x-MnO_2_ (mineral of unknown origin), and (3) Psilomelan (Minas Gerais – Brazil). The three samples were used to characterize the impact of differences in MnO_2_ intrinsic reactivity. Manganit was the quantitatively more abundant mineral available and was used in all experiments, while x-MnO_2_ and Psilomelan were used only in parallel comparative experiments. The natural minerals were crushed and fractionated by sieving. The fraction 0.5–1.0 mm was used without any further pre-treatment. No chemical, mineralogical nor structural characterizations were performed. MnO_2_ is a reactive mineral^33,59^ and is used to delay the availability of ‘free’ iron corrosion products (FeCPs) in the system. This results in a delay of quantitative MB discoloration^28,35^. Using three different natural MnO_2_ minerals intended to validate the premise that each material has it own intrinsic reactivity^33^.

#### Sand

The used sand was a commercial material for aviculture (“Papagaiensand” from RUT – Lehrte/Germany). The sand was used as received without any further pre-treatment. The particle size was between 2.0 and 4.0 mm. Sand was used as an adsorbent because of its worldwide availability and its use as admixing agent in Fe^0^ barriers^60,61^. The adsorption capacity of sand for MB has been systematically documented as early as in 1955 by Mitchell et al.^53^.

### MB discoloration

Quiescent batch experiments (non-shaken) were conducted in assay tubes for experimental durations of up to 60 d. The batches consisted of 0.0 to 1.0 g of sand, 0.0 to 1.0 g of Fe^0^, 0.0 to 1.0 g of MnO_2_ and mixtures thereof in 22.0 mL of a 10.0 mg L^–1^ MB solution. The investigated systems were: (1) Fe^0^ alone, (2) sand alone, (3) MnO_2_ alone, (4) Fe^0^/sand, (5) Fe^0^/MnO_2_ and (6) Fe^0^/sand/MnO_2_. The efficiency of individual systems at discolouring MB was characterized at laboratory temperature (about 22° C). Initial pH was about 8.2. After equilibration, up to 3.0 mL of the supernatant solutions were carefully retrieved (no filtration) for MB measurements (no dilution). Each experiment was performed in triplicates, and averaged values are presented.

Three different MB discoloration experiments were conducted: (1) in Fe^0^/sand systems for 3 to 60 d; (2) in Fe^0^_i_/sand/MnO_2_ for 47 d (varying Fe^0^ loading); and (3) in Fe^0^/sand/MnO_2i_ for 47 d (varying MnO_2_ loading). The subscripts ‘i’ refers to the aggregate which mass loading is varying (Fe^0^ or MnO_2_). Table 1 summarizes the aggregate content of the 7 Fe^0^/MnO_2_/sand systems investigated herein. The operational reference (blank experiment) is also added. Note that the pure Fe^0^ system (Fe^0^ alone) is regarded as a ‘Fe^0^/MnO_2_/sand system’ without MnO_2_ nor sand.

**Table 1:** Overview on the seven (7) investigated systems.

System	Fe^0^	Sand	MnO_2_	Materials	Comments
	(g L^−1^)	(g L^−1^)	(g L^−1^)		
Reference	0.0	0.0	0.0	none	Blank experiment
System 1	4.5	0.0	0.0	Fe^0^ alone	Blank for Fe^0^
System 2	0.0	25.0	0.0	sand alone	Blank for sand
System 3	0.0	0.0	2.3	MnO_2_ alone	Blank for MnO_2_
System 4	4.5	25.0	0.0	Fe^0^/sand	Reference system
System 5	4.5	0.0	4.5	Fe^0^/MnO_2_	Reference system
System 6	0.0	25.0	4.5	Sand/MnO_2_	None
System 7	2.3 to 45	25.0	2.3 to 45	Fe^0^/sand/MnO_2_	Fe^0^ or MnO_2_ as variable

**Figure 1:**
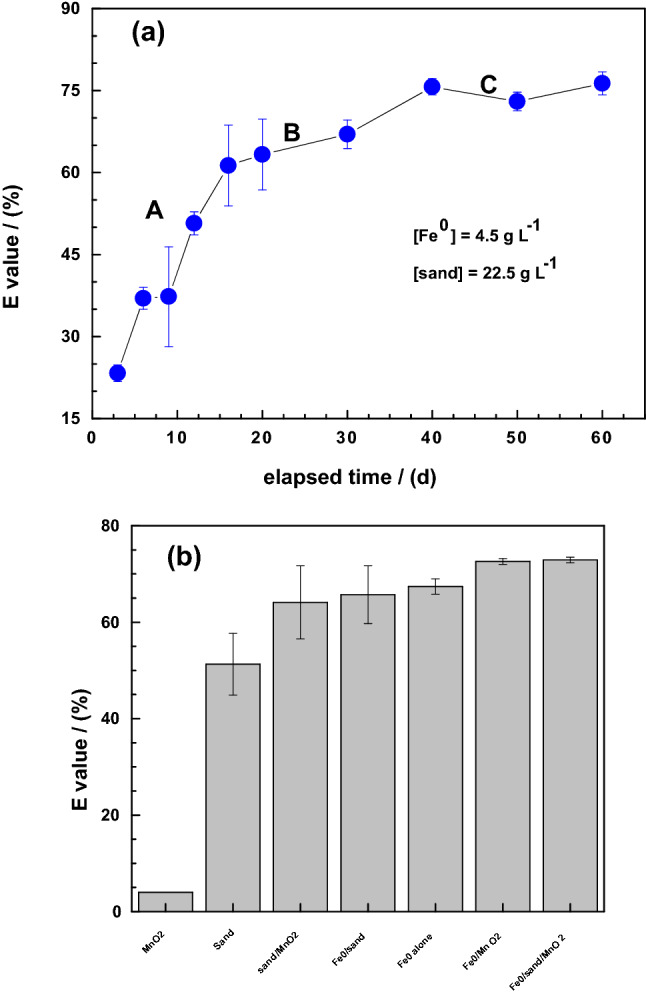
Comparison of the efficiency of tested materials for methylene blue (MB) discoloration: (**a**) for 0 to 60 days in Fe0/sand system, and (**b**) by the tested systems for 47 days. Experimental conditions: [Fe^0^] = 2.3 to 45 g L^−1^ in (**a**), 4.5 g L^−1^ in (**b**); [sand] = 45 g L^−1^; and [MnO_2_] = 2.3 g L^−1^ in (**a**) and 4.5 g L^−1^ in (**b**). The lines are not fitting functions, they simply connect points to facilitate visualization.

The material loadings correspond to Fig. 1b.

### Analytical methods

Iron and MB aqueous concentrations were determined by a Cary 50 UV–Vis spectrophotometer (Varian). The working wavelengths for MB and iron were 664.5 and 510.0 nm, respectively. Cuvettes with 1.0 cm light path were used. The spectrophotometer was calibrated for Fe and MB concentrations ≤ 10.0 mg L^−1^. The pH value was measured by combined glass electrodes (WTW Co., Germany).

### Expression of MB discoloration results (E value)

In order to characterize the magnitude of the tested systems for MB discoloration, the discoloration efficiency (E) was calculated (Eq. 3). After the determination of the residual MB concentration (C), the corresponding percent MB discoloration (E value) was calculated as:

3 $$ {\text{E = }}[1 - ({\text{C}}/{\text{C}}_{0} )]*100\% $$

where C_0_ is the initial aqueous MB concentration (ideally 10.0 mg L^−1^), while C gives the MB concentration after the experiment. The operational initial concentration (C_0_) for each case was acquired from a triplicate control experiment without additive material (so-called blank). This procedure was to account for experimental errors during dilution of the stock solution, MB adsorption onto the walls of the reaction vessels, and all other possible side reactions during the experiments.

## Results and discussion

### Evidence for chemical reactions

#### Triphasic MB discoloration in the Fe^0^/sand system

Figure 1a shows a triphasic pattern in the process of MB discoloration in the Fe^0^/sand system. The initial discoloration (up to day 15) is very rapid (phase A), followed by slower discoloration between days 16 and 35 (phase B), and a plateau for the rest of the experimental duration (d > 35) (phase C). It can be considered that after 35 days, a pseudo-equilibrium stage is achieved. This stage is characterized by the complete coverage of sand by FeCPs such that further MB discoloration solely results from adsorption and co-precipitation with free FeCPs (Table 2). Herein, “free” operationally characterizes (not quantifies) the fraction of FeCPs which precipitates after the sand surface is completely coated. This means that, if the impact of MnO_2_ on the Fe^0^/H_2_O should be characterized under the named operational conditions, experiments should last for more than 35 days. Based on this observation, the impact of MnO_2_ was investigated for an equilibration time of 47 days. Note that the absolute E values from the data in Fig. 1 and the rest of the work are not necessarily directly comparable because the experiments were not performed in parallel. Although the Fe^0^ material was from the same supplier, the reactivity of a Fe^0^ specimen also depends on it surface state, which depends on the storing conditions and the duration of storage^21,56,57,62^. The experiments yielding the results in Fig. 1a were performed more than 12 months^18^ after the other experiments^35^. The objective was to understand why results of MB discoloration experiments for 14 days^35^ and 47 days^28^ in ternary systems (Fe^0^/MnO_2_/sand) vary widely.

**Table 2:** Time dependent inventory of reactive species in the four investigated systems.

System	Fe^0^	MnO_2_	Sand	Fe^0^/MnO_2_/sand
t_0_ = 0	Fe^0^	MnO_2_	Sand	Fe^0^ + MnO_2_ + Sand
t > t_o_	Fe^0^ + FeCPs	MnO_2_ + Mn^2+^	Sand	Fe^0^ + MnO_2_ + Sand + FeCPs + Mn^2+^
t_¥_	FeCPs	Mn^2+^	Sand	Mn^2+^ + Sand + FeCPs

t_0_ corresponds to the start of the experiment, while t_¥_ corresponds to Fe^0^ depletion. It is assumed that MnO_2_ is converted to Mn^2+^ without impact on MB discoloration. FeCPs = Fe corrosion products. FeCPs can be free or coated on sand.

Figure 1b summarizes the extent of MB discoloration in the seven possible systems (Table 1): (1) three single-aggregates (Fe^0^, MnO_2_, sand), (2) three binary-aggregates (Fe^0^/sand, Fe^0^/MnO_2_, sand/MnO_2_), and (3) the ternary Fe^0^/sand/MnO_2_ system. The uniqueness of the single-sand MnO_2_ (100% MnO_2_) relative to the other systems is that it contains no in-situ generated FeCPs (Table 2) and is not a good adsorbent for MB according to its low points of zero charge^63^. It is seen that Fe^0^-based systems performed better than single and binary systems with sand and MnO_2_ for the selected equilibration time (47 d). For the Fe^0^-based systems, those containing MnO_2_ performed the best. While this observation contradicts the report of Miyajima and Noubactep^35^, it is consistent with literature reports^40,41,44,45^. Because MnO_2_ alone had the least E value (only 4%), the contribution of this mineral for MB discoloration is indirect and coupled to the presence of Fe^0^.

#### Dissolved iron in the investigated systems

The results of iron release are not shown herein. In Fe^0^/sand and Fe^0^/sand/MnO_2_ systems, the iron level was constantly lower than 0.2 mg L^–1^ as the contact time varies from 3 to 60 d. This observation is rationalized by: (1) the progressive in-situ coating of sand in the Fe^0^/sand, (2) sand coating and Fe^2+^ consumption in the Fe^0^/sand/MnO_2_ system. Note that, measured dissolved iron is the balance between Fe^0^ oxidative dissolution (Fe source) and (3) all processes consuming Fe (Fe sinks). Fe sinks include: (1) Fe adsorption onto minerals (FeCPs, MnO_2_ and sand), (2) iron precipitation as hydroxides, and (3) Fe^2+^ utilization for the reductive dissolution of MnO_2_^21,35,43,64^. Thus, the ternary system (Fe^0^/sand/MnO_2_) has one Fe sink more than the Fe^0^/sand system. This is the major reason of the observed absence of Fe release. Previous works testing the pure Fe^0^ system in parallel reported on [Fe] > 0.2 mg L^−1^ for contact time lower than 20 days^35^. Similar low Fe levels were reported by Gatcha-Bandjun et al.^64^ in quiescent batch experiments for up to 60 days. This observation recalls that, at pH > 4.5, the dissolved Fe level can only support the interpretation of achieved results^65^. This observation also reiterates the complex nature of the Fe^0^/H_2_O system and the crucial importance of long-term experiments under various operational conditions^21,28,65,66^ for a better understanding. Remember that the addition of sand and MnO_2_ is a tool to better characterize the Fe^0^/H_2_O system as a whole under natural-near conditions.

### MB discoloration in Fe^0^/sand/MnO_2_ systems

**Figure 2:**
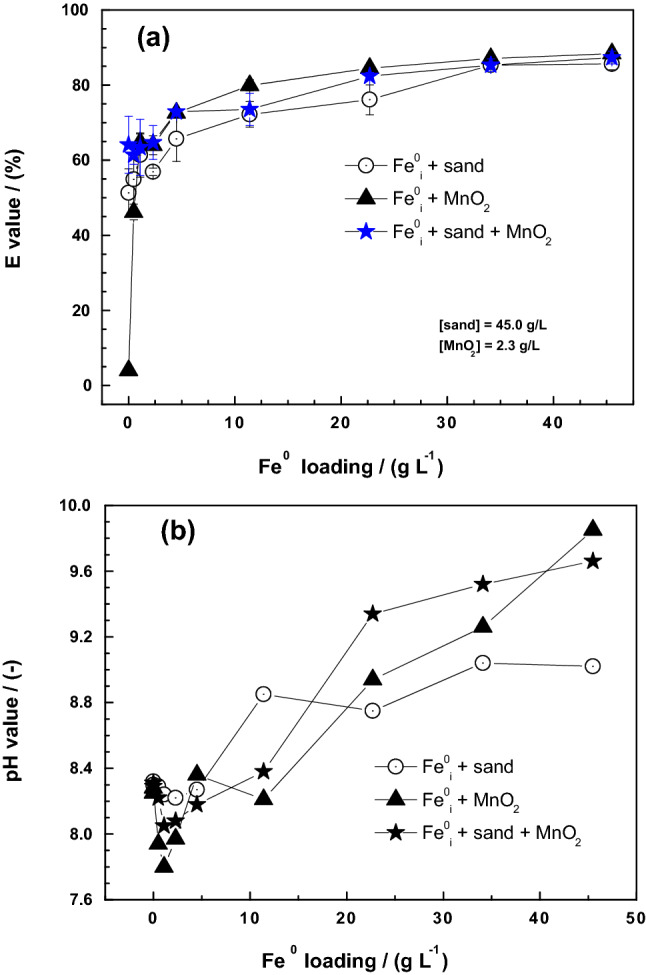
Changes in the Fe^0^, Fe^0^/sand, and Fe^0^/sand/MnO_2_ systems as impacted by the addition of various Fe^0^ loading for 47 days: (**a**) Methylene blue discoloration, and (**b**) pH value. Experimental conditions: [Fe^0^] = 2.3 to 45 g L^−1^; [sand] = 22.5 g L^−1^; and [MnO_2_] = 2.3 g L^−1^. The lines are not fitting functions, they simply connect points to facilitate visualization.

Figure 2a shows a biphasic pattern in the process of MB discoloration in the three systems with the Fe^0^/MnO_2_ being the most interesting. In the sand-based systems, MB discoloration at [Fe^0^] = 0 g L^−1^ (> 50%) is explained by the strong adsorption affinity of sand for MB^35,53^. This means that the observed enhanced MB discoloration due to Fe^0^ and MnO_2_ is explained by the continuous corrosion beyond sand coating, as free precipitation of FeCPs becomes possible. MB is adsorbed onto sand and/or precipitated with FeCPs, but note that is a slow process occurring on a “passivated Fe^0^” (“reactivity loss”). In this study, the investigation of “residual” reactivity is rendered possible by in-situ coating of sand and Fe^2+^ oxidation by MnO_2_ (Eq. 2). Both processes delay the “passivation” of Fe^0^.

In the binary Fe^0^/MnO_2_ system, MB discoloration occur solely due to Fe^0^ oxidation and the subsequent precipitation of FeCPs which eliminate MB from the aqueous solution. Under the test conditions, 47 d were long enough to enable in-situ generation of enough FeCPs for MB discoloration to an extent larger that in the binary Fe^0^/sand and the ternary Fe^0^/sand/MnO_2_ systems (Fig. 2a). It can be considered that sand is a confounding factor in the kinetics of MB discoloration. However, without sand, Fe^0^ particles are cemented to each other and become less or non-reactive^67^. Note that sand is a central component for the MB method^35,56–58^. As seen in Fig. 1b, MnO_2_-based systems are the most efficient systems in the long-term. Accordingly, Fe^0^/sand/MnO_2_ systems are more efficient than Fe^0^ and Fe^0^/sand because of the sustained Fe^0^ reactivity. The discussion herein suggests that binary Fe^0^/MnO_2_ systems are more efficient than Fe^0^/sand because besides being non-expansive, MnO_2_ is also reactive (Eq. 2).

Figure 2b shows changes of the pH value in the three systems as the Fe^0^ loadings increase from 0 to 45 g L^−1^. The initial pH value for all systems was 8.2. It is evident that there is a general increase in pH value with increasing Fe^0^ loading. The fact that there is a slight pH decrease for lower Fe^0^ loading ([Fe^0^] < 7.5 g L^−1^) is reproducible and has been observed by other researchers^67^. This shows that for these low Fe^0^ loadings, iron corrosion according to Eq. (1) has not dominated concurrent processes like SiO_2_ dissolution (acidifying). For [Fe^0^] < 7.5 g L^−1^, sustained iron corrosion is clearly reflected even though: (1) no clear difference is observed in the extent of MB discoloration, and (2) no Fe was detected in the aqueous phase (data not shown). Again, the MnO_2_-bearing systems depicted the highest pH increase, corroborating the fact that MnO_2_ enhances the efficiency of Fe^0^/H_2_O systems by sustaining Fe^0^ corrosion (Eq. 2)^32,39,45^. It is expected that a different Fe^0^ specimen, or a different loading of the same specimen will reproduce the trend observed herein. For example, while using two different mass loadings of the same Fe^0^ specimen, Touomo-Wouafo et al.^68^ reported on different pics in the concentration of aqueous Fe^2+^. In fact, the corrosion rate of Fe^0^ materials varies largely under environmental conditions^29,30,62,69^. Unfortunately, despite 30 years of intensive research on the application of Fe^0^ materials for environmental remediation, no single standardized/unified protocol for the characterization of the material intrinsic reactivity has been presented^69–72^. The next section compares the behavior of the same Fe^0^/sand system as the mass loading of three different natural MnO_2_ specimens vary from 0 to 45 g L^−1^.

### The impact of the different MnO_2_

**Figure 3:**
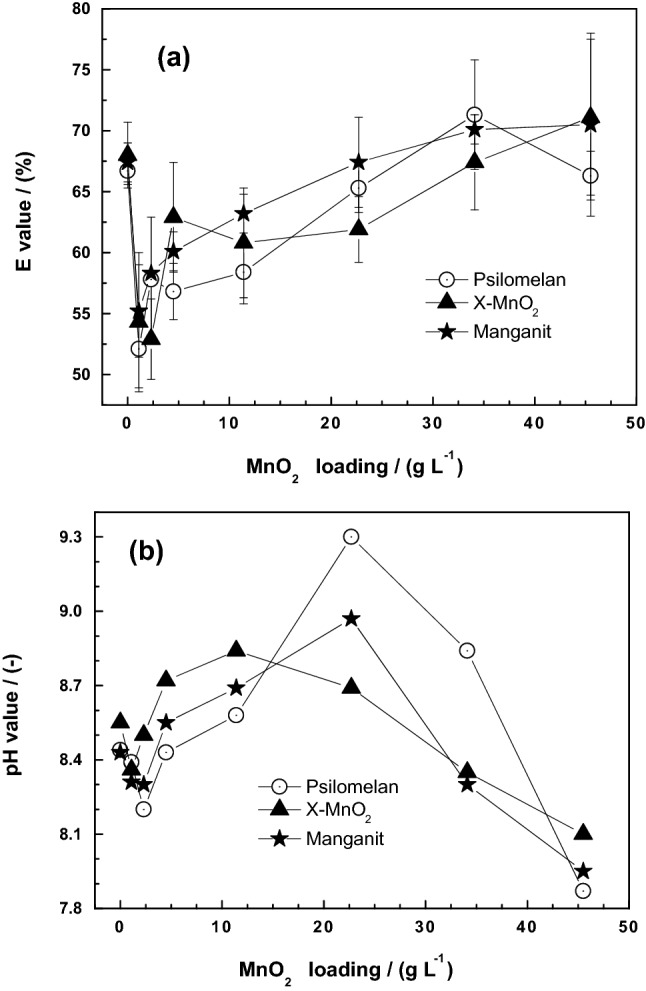
Changes in the Fe^0^/sand/MnO_2_ systems as impacted by the addition of various MnO_2_ loading for 47 days: (**a**) Methylene blue discoloration, and (**b**) pH value. Three different natural minerals were tested. Experimental conditions: [Fe^0^] = 4.5 g L^−1^; [sand] = 22.5 g L^−1^; and [MnO_2_] = 2.3 to 45.0 g L^−1^. The lines are not fitting functions, they simply connect points to facilitate visualization.

Figure 3a shows changes of the extent of MB discoloration by the Fe^0^/sand system as the loading of three different natural MnO_2_ varies from 0 to 45 g L^−1^. The E value for [MnO_2_] of 0 g L^−1^ corresponds to MB discoloration by the Fe^0^/sand system which is about 67%. This value decreases to about 55% for all three MnO_2_ specimens for the lowest mass loading ([MnO_2_] = 2.3 g L^−1^) and subsequently increase with increasing MnO_2_ loading. The increase of the E value is not linear but monotonous, revealing the complexity of processes in the ternary-aggregate systems. It is seen that Manganit performed better than the two other MnO_2_ specimens. The two most important features from Fig. 3a are: (1) MnO_2_ enhances the efficiency of Fe^0^/H_2_O systems, and (2) each MnO_2_ specimen has its own dissolution kinetics (intrinsic reactivity). Considering that the redox reactivity of contaminants is not considered in the presentation until now, it becomes clear that the semi-conductive nature of MnO_2_ and its redox reactivity for selected contaminants need to be properly addressed in the remediation context.

Figure 3b shows changes of the pH value in the three systems as the MnO_2_ loadings increase from 0 to 45 g L^−1^. The pH of the Fe^0^/sand system ([MnO_2_] = 0 g L^−1^) increased from an initial value of 8.2 to about 8.5 after 47 days of equilibration. For the lowest tested MnO_2_ loading, the pH value decreased from 8.2 to about 7.8, then subsequently increased to reach a maximum of 8.9 around [MnO_2_] = 25 g L^−1^. Thereafter, the pH value decreased to about 7.9 for [MnO_2_] = 45 g L^−1^. The largest pH variation was observed with Psilomelan while the behavior of the two other minerals were very close to each other. In the absence of the mineralogical composition of the MnO_2_ specimens, these observations cannot be further interpreted. It is however certain that: (1) for higher MnO_2_ loading, the final pH value was not determined by iron corrosion, and (2) each MnO_2_ mineral is a stand-alone operation parameter. Clearly the results presented herein are highly qualitative. Michel et al.^33^ recently advocated for the introduction of procedure standardizing the reactivity of manganese oxides (Mn_x_) for water treatment.

### Discussion

#### Promoting iron corrosion under natural conditions

The main result of this work is that by scavenging Fe^2+^ from Fe^0^ oxidative dissolution, MnO_2_ minerals intensify the process of decontamination. In other words, the reductive dissolution of MnO_2_ (Eq. 2) sustains iron corrosion (Eq. 1) according to Le Chatelier principle. Sand is used to sustain the discussion in the framework of the used MB method but has the practical significance of being the most available aggregate to build Fe^0^-based reactive zones^24,73^. This result leads to several avenues for addressing both the reactivity loss and the permeability loss of Fe^0^ filters. As for addressing the “reactivity loss” the experiments presented herein, quiescent batch experiments for 47 days, were performed under conditions corresponding to a “passivated” state for Fe^0^. Nevertheless significant changes within the systems (e.g., pH value) could be documented. It shall be recalled that MB is an indicator of reactivity and that using a species with strong affinities to FeCPs (e.g., Orange II or methylorange) would have enabled more differentiation between the systems^64,74–76^. In essence, by combining the most suitable Fe^0^ and MnO_2_ a large array of appropriate remediation systems for site-specific applications can be designed. As far as filtration systems are concerned, beside the nature and the extent of pollution and the required quality of treated water, relevant operation parameters include: (1) the Fe^0^ nature (e.g., form, reactivity, size), (2) the MnO_2_ nature and reactivity, (3) the Fe^0^/MnO_2_ mixing ratio (including in composites), (4) the exact amount of each material (e.g. mass or volume), (5) the size of the filter, (6) the flow velocity (e.g. residence time), and (7) depth of the filter. More research is needed to enable the realization of the huge potential of the Fe^0^/MnO_2_ system for water treatment.

#### Significance of the results in the design and operation of Fe^0^/H_2_O systems

The common underlying mechanism for water decontamination using Fe^0^ is its electrochemical oxidative dissolution by protons and by protons alone^66,77^. This century old knowledge has been distorted while introducing the Fe^0^ remediation technology in the 1990s^78^. Since then, researchers are desperately seeking for ways to establish reliable non-site-specific criteria for the design of Fe^0^-based systems^58,79^. If such design criteria are established, then, site-specific treatability studies may only be required to fine-tune design criteria for the optimal performance of Fe^0^ systems^79^. Unfortunately, all data provided in the framework of these efforts have not really helped and a clear circular reasoning is established within the Fe^0^ remediation research community^58,80^. Clearly, numerous laboratory, pilot, and field-scale studies for water treatment by Fe^0^ have just demonstrated the feasibility of the technology, but the science behind is yet to be established. The results of the study are regarded as a decisive contribution on the path to establish the science of the Fe^0^/H_2_O system.

Since the seminal work of Schreier and Reinhard^81^ describing a lag time between Fe^0^ immersion and the start of contaminant “reductive transformation”, many arguments have been advanced to rationalize this observation, the most prominent being that is corresponds to the time to reduce atmospheric corrosion products, Fe^III^ oxides and hydroxides^82–84^. However, these earlier arguments should be collectively regarded as wrong because if contaminant reduction was occurring at the Fe^0^ surface, it should be quantitative when this surface is free, that is immediately after immersion^45,64,68,85–87^. Interested readers are referred to the cited literature, in particular Touomo-Wouafo et al.^68,86^ using polarography to follow changes in Fe^2+^ and metallic ions (including Zn^2+^) of polluted waters. Touomo-Wouafo et al.^68^ described an induction phase following Fe^0^ immersion, followed by a reactive phase during which Fe^2+^ appears in solution and Zn^2+^ is removed. The reactive phase was followed by a passivation phase characterized by no detection of Fe^2+^ in solution and insignificant Zn^2+^ removal. The experiments of Touomo-Wouafo et al.^68^ lasted for up to 16 days under buffered conditions. The first merit of the present work is to have extended the reactive phase beyond 16 days, while demonstrating that despite the absence of Fe^2+^ in the bulk solution, there is no passivation. In other words, the system is still reactive and will maintain reactivity as long as Fe^0^ is not completely depleted. The research question is thus: How to sustain the residual Fe^0^ corrosion to the extent that satisfactorily water treatment is achieved in the long-term? A qualitative answer from the results achieved herein is “add MnO_2_”. The next step is to pilot test this idea while keeping in mind that each Fe^0^ and MnO_2_ is a different reactive material that require prior characterization before use^38,69^. Only systematic investigations with well-characterized reactive (e.g. Fe^0^ and MnO_2_) and non-reactive aggregates (e.g., gravel, pumice, sand) will enable the design of more efficient and sustainable MnO_2_-amended Fe^0^/H_2_O systems.

#### The role MnO_2_ in the context of the inconsistencies in Fe^0^ literature

Decentralized wastewater treatment and safe drinking provision is increasingly using hybrid Fe^0^-based filtration systems. Commonly used mineral materials are anthracite, diatomite. gravel, magnetite (Fe_3_O_4_), manganese ores (MnO_x_), pumine, quartz, sand and zeolite^38,88^. From these minerals quartz and sand are most commonly used and are considered "neutral" filter media^61,67,73,89^. In this context, neutral means non-reactive or inert. In the framework of Fe^0^ filters, the non-expansive nature of sand is already very important as Fe^0^/sand filters are more sustainable than pure Fe^0^ filters^24,25,43^. The operating mode of sand in sustaining Fe^0^/sand filters relies on the evidence that sand is in-situ coated with iron oxyhydroxides which spatially precipitate far from the Fe^0^ surface, thereby delaying its passivation (‘reactivity loss’). The contribution of sand to the sustainability of Fe^0^ filters arises from it inert nature making it a non-expansive aggregate and retarding clogging compared to a pure Fe^0^ filter^25^. Apart from quartz and sand, other minerals are considered to have some adsorptive (and redox) affinities to many dissolved species and are not employed as pure filling material. The two most investigated minerals in the context of Fe^0^ filters are Fe_3_O_4_^88^ and FeS_2_^58^.

To the best of authors’ knowledge, manganese ores (MnO_x_) have been introduced as means to investigate the mechanism of uranium removal in Fe^0^/H_2_O systems^90,91^. The achieved results inspired Burghardt and Kassahun^39^ to test a Fe^0^/MnO_2_ reactive zone for the removal of radium and uranium from groundwater. Further investigations of the Fe^0^/MnO_2_ were performed around 2010 by two different research groups: Dr. Ghauch in Beirut/Lebanon^41,42^, and Dr. Noubactep in Göttingen/Germany^32,42,51,55^. Their results unequivocally confirmed enhanced contaminant removal by Fe^0^/MnO_2_ relative to pure Fe^0^ systems. This observation was justified by the ‘reinforcement’ of the corrosion process by the reductive dissolution of MnO_2_ or better MnO_x_. From more recent reports available in the scientific literature, only the research group of Dr. Gheju in Timisoara/Romania has designed his investigations based on this past knowledge. All other researchers have not considered this decades-old knowledge while others even distort it. The most representative example is perhaps Dong et al.^47^ who investigated the MnO_2_/Fe^0^ sequence (Fe^0^ after MnO_2_) and discussed their results as if the aggegates were mixed in the same layer. In a effort to build a common knowledge database for Fe^0^-based remediation systems, it is very disappointing that new experiments are not designed based on available knowledge and their interpretation ignored them as well. In the past two decades, the importance of the integrity of science has gained the due importance^92–95^. There will be no progress on this path until authors consider their personal integrity. This proliferation of scientific misconduct^92,94^ suggest that, beyond the current metrics (e.g., h-index, number of citations) individual researchers have to be evaluated based on their personal integrity. It is at least certain that while cheating the editors and reviewers with wrong novelties, publications contrary to the state-of-the-art knowledge confuse early-career researchers, including PhD candidates^80,96,97^.

The present study has reinforced the view that water treatment in Fe^0^ filters is characterized by the in-situ generation of iron oxyhydroxides (FeCPs) and their retention in the filter bed. FeCPs are excellent scavengers of both biological and chemical contaminants^5,85,98–100^. Because oxyhydroxides are larger in volume than the parent metal (Fe^0^), Fe^0^/sand filters are more sustainable than pure Fe^0^ filters. Yet if sand is partly or completely replaced by MnO_2_, in addition to ‘creating’ space for generated FeCPs, the corrosion process is ‘reinforced’ or the passivation delayed^51^. By demonstrating this in the present paper with an indicator of reactivity for the Fe^0^/H_2_O system (MB), its universality is proved. The remaining task is to characterize both Fe^0^ and MnO_2_ and determine their ratio in site-specific applications. That means seeking for knowledge of surface morphology of Fe^0^ and MnO_2_ or their time-dependant changes. The next section outlines a possible research program.

#### Designing MnO_2_-amended Fe^0^ filters

MnO_2_-amended Fe^0^ filters are a particular case of Fe^0^ filters in which the reduced oxidation kinetics of Fe^0^ (‘reactivity loss’) is reinforced by the addition of MnO_2_. MnO_2_ addition ultimately increases the service life of the Fe^0^ filter^39^. Despite the knowledge of the operating mode of MnO_2_ to enhance the efficiency of Fe^0^ filters, little is understood about the intrinsic reactivity of both materials, and how they behave in the long-term^38,69,72^. Moreover, limited data exist on the reactivities of the various forms of MnO_2_, including those invested in the present study^38^. There are numerous Fe^0^ and MnO_2_ suppliers around the world and each individual material is a stand-alone operational variable for a MnO_2_-amended Fe^0^ filter. Therefore, the major research question for the next-generation Fe^0^ filters is: How are existing chemical, physical and structural differences between available and/or newly manufactured Fe^0^ and MnO_2_ affecting their efficiency for water treatment? To answer this research question, the major research objectives for the coming filters are as follows:

1. To characterize the available Fe^0^ and MnO_2_ in terms of the chemical, compositional and physical properties, and identify the structure of each material;
2. To characterize the available Fe^0^ for their intrinsic reactivity in aqueous solutions using available tools^69,72^;
3. To develop and validate tools to characterize the intrinsic reactivity of MnO_2_ in aqueous solutions;
4. To analyse and compare the performance of various Fe^0^ and MnO_2_ (and their mixtures) for MB disccoloration and for the removal of selected model contaminants from aqueous solution using long-lasting fixed-bed columns (> 6 months);
5. To investigate changes in contaminant removal performance when different Fe^0^/MnO_2_ ratios are used;
6. Finally, to synthesize serviceable column media comprising of desired Fe^0^/MnO_2_ mixtures for any site-specific application.

On a positive note, with regard to frugal technologies, apart from Objective 1 (structural characterization), research to address these objectives does not require sophisticated laboratory analytical equipment, and can be implemented with limited research budgets. Thus, all other objectives can be addressed in low-equipped laboratories including those in the developing world^52^.

## Concluding remarks

The concept that aqueous contaminant removal in the presence of metallic iron (Fe^0^/H_2_O system) is caused by the process of Fe precipitation is consistent with many experimental observations. In particular, by delaying Fe precipitation in the bulk solution, MnO_2_ delays the removal process at local-grain scale. However, because in a Fe^0^ bed this process occurs thousands of times (filter scale), the presence of MnO_2_ is favourable for the sustainability of Fe^0^/H_2_O systems. In fact, without MnO_2_, “Fe^0^ passivation” occurs earlier and the Fe^0^/H_2_O system may fail despite abundance of Fe^0^. The present study has used the MB method to demonstrate this elegantly. Past efforts to rationalize the operating mode of MnO_2_ amended Fe^0^/H_2_O systems were challenging also because biotic and/or abiotic interactions of relevant contaminants with both Fe^0^ and MnO_2_ were to be considered.

The merit of this study is to have identified a reaction time (> 35 d) and experimental conditions (quiescent bath with the given material loadings) under which the made demonstration was possible. The presentation was limited at highlighting the key result: Fe^0^ generates Fe minerals which interact with sand and MnO_x_ to treat water. Exploiting this knowledge to design more efficient and sustainable Fe^0^/H_2_O systems goes through systematic investigations. The chemistry, mineralogy, morphology, and structure of both Fe^0^ and MnO_x_ affect the results of water treatment. Their relative amounts and proportions in filters as well as the water chemistry are other equally important variables. This multitude of inter-dependent factors makes a systematic approach mandatory if comparable and transferable results are sought. In other words, thoroughly planed experiments, designed variously with well-characterized materials and using controlled flow conditions are urgently necessary to make Fe^0^ filtration a sort of “best available technology” among appropriate technologies for decentralized water treatment.
